# Effect of low- and high-carbohydrate diets on swimming economy: a crossover study

**DOI:** 10.1186/s12970-020-00392-3

**Published:** 2020-12-09

**Authors:** Merry A. Bestard, Jeffrey A. Rothschild, George H. Crocker

**Affiliations:** 1 School of Kinesiology, Nutrition & Food Science, California State University, Los Angeles, 5151 State University Dr, Los Angeles, CA 90032 USA; 2 Sports Performance Research Institute New Zealand (SPRINZ), Auckland University of Technology, Auckland, New Zealand

**Keywords:** Fat, Energy expenditure, Efficiency, Macronutrients

## Abstract

**Background:**

Swimming economy refers to the rate of energy expenditure relative to swimming speed of movement, is inversely related to the energetic cost of swimming, and is as a key factor influencing endurance swimming performance. The objective of this study was to determine if high-carbohydrate, low-fat (HCLF) and low-carbohydrate, high-fat (LCHF) diets affect energetic cost of submaximal swimming.

**Methods:**

Eight recreational swimmers consumed two 3-day isoenergetic diets in a crossover design. Diets were tailored to individual food preferences, and macronutrient consumption was 69–16-16% and 16–67-18% carbohydrate-fat-protein for the HCLF and LCHF diets, respectively. Following each 3-day dietary intervention, participants swam in a flume at velocities associated with 50, 60, and 70% of their maximal aerobic capacity (VO_2max_). Expired breath was collected and analyzed while they swam which enabled calculation of the energetic cost of swimming. A paired t-test compared macronutrient distribution between HCLF and LCHF diets, while repeated-measures ANOVA determined effects of diet and exercise intensity on physiological endpoints.

**Results:**

Respiratory exchange ratio was significantly higher in HCLF compared to LCHF (*p* = 0.003), but there were no significant differences in the rate of oxygen consumption (*p* = 0.499) or energetic cost of swimming (*p* = 0.324) between diets. Heart rate did not differ between diets (*p* = 0.712), but oxygen pulse, a non-invasive surrogate for stroke volume, was greater following the HCLF diet (*p* = 0.029).

**Conclusions:**

A 3-day high-carbohydrate diet increased carbohydrate utilization but did not affect swimming economy at 50–70% VO_2max_. As these intensities are applicable to ultramarathon swims, future studies should use higher intensities that would be more relevant to shorter duration events.

## Introduction

It is well established that providing adequate carbohydrate to working muscles is a key contributor to optimal endurance performance [1]. However, there has been an increased interest in low-carbohydrate, high-fat (LCHF) diets in recent years, as a mechanism to increase fat oxidation during exercise and utilize more of the body’s fat stores [2]. A high capacity for fat oxidation may be particularly important for athletes competing in ultra-distance events [3], and adaptation to a LCHF diet could benefit long-distance swimmers who perform prolonged sub-maximal exercise with limited opportunity to consume adequate carbohydrate [2, 4].

Broadly, two iterations of LCHF dietary approaches exist – ketogenic (providing < 5% of energy intake from carbohydrate) and non-ketogenic (providing ~ 15–20% of energy intake from carbohydrate) [5]. A non-ketogenic LCHF diet can be used as part of a nutritionally periodized dietary plan featuring periods of reduced carbohydrate intake in order to maximize fat-burning adaptations [5]. Approximately 20% of endurance athletes follow a LCHF or periodized carbohydrate dietary pattern, the majority of whom also perform fasted-state training related to a desire to increase fat oxidation during exercise [6].

Despite an increased capacity for fat oxidation, a LCHF diet may impair movement economy [7, 8]. Movement economy refers to the rate of energy expenditure relative to speed of movement (i.e., cycling, running, or swimming), and has been identified as a key factor influencing endurance sport performance [9]. It is a multifactorial phenomenon reflecting various metabolic, cardiorespiratory, biomechanical, and neuromuscular characteristics of an athlete [10]. Economy is calculated from the rate of energy expenditure and velocity during steady-state, submaximal exercise. Because the oxidation of carbohydrate results in a greater caloric value per liter of O_2_ consumed compared with fat oxidation [11], it is possible that movement economy can be influenced by changes in substrate utilization. Indeed, improved cycling efficiency (i.e., lower rate of energy expenditure at the same power) has been reported following 3 days of a high-carbohydrate, low-fat (HCLF) diet [12], and movement economy was decreased (i.e.*,* higher rate of energy expenditure at the same velocity) in runners and race walkers following 5 days [7, 8] and 3–4 weeks [13–15] of ketogenic LCHF dietary interventions. It is currently unknown if dietary changes can influence swimming economy.

Swimming economy may be a strong predictor of success in long-distance swims such as the 10-km marathon and ultramarathon (> 10 km) events [4]. Economy may have a greater influence on swimming performance compared to cycling or running economy because swimmers move through water, a fluid 700–800 times denser than air. Factors known to affect swimming economy include age, sex, training status, body size, hydrostatic lift, and torque [16–18]. While economy during running and cycling is commonly measured via indirect calorimetry, it has traditionally been difficult to collect a swimmer’s expired breath due to logistical constraints. However, the use of a swim flume allows control of a swimmer’s velocity and enables metabolic measurements to be obtained by keeping the swimmer stationary.

To our knowledge, the effect of diet on swimming economy has not been studied. Therefore, the aim of this study was to determine the influence of 3 days of high- and low-carbohydrate diets on the energetic cost of submaximal swimming. We hypothesized that a high-carbohydrate diet would result in better swimming economy (i.e., lower energetic cost of swimming; C_s_) related to an increased respiratory exchange ratio (RER) and, therefore, greater energy conversion per volume of oxygen consumed when utilizing carbohydrates compared to fats.

## Methods

### Experimental overview

**Fig. 1:**
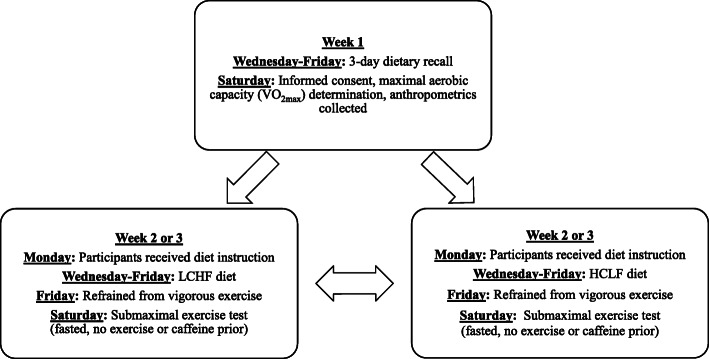
Schematic of study design. VO_2max_ = maximal aerobic capacity; HCLF diet = high-carbohydrate, low-fat diet; LCHF = low-carbohydrate, high-fat diet

This study used a randomized, crossover design with participants reporting to the laboratory on 3 days (Fig. 1). The first visit consisted of an incremental swimming test to volitional fatigue in a swim flume to determine maximal aerobic capacity (VO_2max_). The second and third visits consisted of submaximal swimming at velocities equivalent to 50, 60, and 70% of VO_2max_ as determined during the first visit. Prior to the second and third visits, participants consumed either a HCLF or a LCHF diet for 3 days. A non-ketogenic LCHF diet was chosen a) to study its use as the type of short-term dietary change that would be included in a periodized nutrition plan, and b) to remove the confounding effect of ketones on the relationship between respiratory exchange ratio and substrate utilization during exercise [19]. Dietary interventions were randomly assigned and separated by a 4-day washout period.

### Participants

Eight healthy, recreational swimmers (4 male, 4 female, 34.6 ± 9.4 years old, BMI 23.8 ± 2.6 kg m^− 2^, VO_2max_ 42.4 ± 8.5 ml kg^− 1^ min^− 1^, average swim training volume 5.2 ± 2.3 h per week) were recruited from a local swim club. To be included in the study, participants had to be between the ages of 18–59 years old, swimming > 3 km per week, and be willing to manipulate their dietary patterns for 2 weeks. Participants were informed of the risks and benefits of participating and provided written informed consent before participating in the study. This study was approved by the California State University, Los Angeles Institutional Review Board (protocol #1419221).

### Procedures

Height and weight were collected on their first visit using a high-capacity column scale (Seca 703, Hamburg, Germany). Participants were instructed to refrain from vigorous exercise for 24 h prior to each visit. All swim tests were completed using a 4.27 × 2.13 m outdoor swimming flume (Endless Pools, Aston, PA, USA). The water depth was maintained at 1.14 m. Water temperature was maintained at 27 °C.

During the first visit, subjects completed an incremental swimming test to exhaustion for determination of VO_2max_. Following a self-selected 5–10 min warm-up in a 22.9-m outdoor pool, participants transitioned to the flume for a 1–2 min familiarization swim. For the graded exercise test, the initial intensity was set at 0.93 m s^− 1^ (1:47 per 100 m) with the speed increasing by 0.09 ± 0.01 m s^− 1^ every 2 min. The test was terminated when participants could no longer maintain pace or when they reached volitional fatigue. Heart rate (HR) was continuously monitored via telemetry (Polar T31, Kempele, Finland). Breath-by-breath gas exchange data were continuously measured using a metabolic cart (Quark CPET; Cosmed, Rome, Italy). The VO_2max_ was determined as the highest 10-s average. The gas analyzers on the metabolic cart were calibrated to ambient air and certified standard gas of known concentration (16% O_2_, 5% CO_2_, 79% N_2_) and gas volume was calibrated with a 3-L syringe.

For the second and third visits participants arrived in a fasted state, with trials performed in the morning at the same time of day following both diet interventions. Participants were allowed a self-selected 5–10 min warm-up in the pool prior to the start of the test. Swimming speeds were established based on the speeds eliciting 50, 60 and 70% of their VO_2max_ during the incremental test on the first visit. The subjects swam at each speed for five minutes and each trial was separated by a brief rest period to drain the snorkel of any collected fluids and provide water ad libitum. Heart rate and breath-by-breath respiratory measurements were continuously collected throughout the exercise trial. The rates of O_2_ consumption (VO_2_) and CO_2_ production (VCO_2_) were determined from the average values over the last two minutes of each 5-min stage. Rate of energy expenditure (EE) was calculated according to the equation of Péronnet & Massicotte [20] and assumed negligible protein oxidation:

Eq. 1 $$ \mathrm{EE}\ \left(\mathrm{J}\ {\mathrm{s}}^{\hbox{-} 1}\right)=281.67\ {\mathrm{VO}}_2\left(\mathrm{L}\ {\min}^{\hbox{-} 1}\right)+80.65\ {\mathrm{VCO}}_2\left(\mathrm{L}\ {\min}^{\hbox{-} 1}\right) $$

The rate of EE was divided by the swimming speed to determine C_s_. Oxygen pulse, a non-invasive estimate of stroke volume was calculated for each participant by dividing VO_2_ by heart rate [21].

### Dietary intervention

Prior to enrollment participants were asked to complete a 3-day dietary food recall on three consecutive weekdays (Wednesday – Friday). Participants were provided with diet record sheets and instructed to accurately record all food and drinks consumed with estimates based on basic household portion sizes. Based on their individual eating behaviors and dietary preferences, individualized HCLF (70% carbohydrate, 15% fat, 15% protein) and LCHF (15% carbohydrate, 70% fat, 15% protein) diets were created using dietary analysis software (ESHA Food Processor Nutrition Analysis, Salem, OR, USA) and provided to each participant. Total energy intake was determined as the average of the Harris-Benedict [22] and Mifflin-St. Jeor [23] resting energy expenditure equations, multiplied by an activity factor of 1.55. Participants received individualized counseling and instruction on how to follow the diets, as well as basic kitchen measuring equipment and a food-safe digital scale (Etekcity, Digital Kitchen Scale, EK6015, Anaheim, CA, USA). Diet instructions were provided on a Monday and participants were told to follow them as closely as possible for the subsequent Wednesday–Friday, before arriving in the fasted-state on Saturday morning for testing. Subjects noted any deviations from their prescribed diets. Seven participants followed the standard protocol with a 4-day washout period between dietary interventions, but due to scheduling conflict one participant had an 11-day washout period.

### Statistical analysis

All data are reported as means and standard deviations. Dietary intake is reported as the average over 3 days and analyzed with a paired t-test, after confirming normality of the data. Repeated measures analysis of variance (2 diets × 3 exercise intensities) determined main effects for diet and exercise intensity on physiological endpoints with Bonferroni corrections used for post hoc testing. The Greenhouse-Geisser correction was used when sphericity was not met. Analyses were performed using Jamovi (Version 1.2.16.0, www.jamovi.org) with statistical significance at *p* <  0.05.

## Results

The macronutrient distribution (carbohydrate-fat-protein) consumed by participants was 69–16-16% for the HCLF group and 16–67-18% for the LCHF group (Table 1). Overall energy intake did not differ between diets (*p* = 0.363). Carbohydrate intake was greater and fat intake was lower (both *p* <  0.001) with the HCLF diet. Protein intake was greater with the LCHF diet (*p* = 0.013).

**Table 1:** Macronutrient intake for the high-carbohydrate, low fat (HCLF) and low-carbohydrate, high-fat (LCHF) diets (*N* = 8)

Diet	Daily energy intake		Carbohydrate			Fat			Protein		
	kcal	kcal kg^**−1**^	g d^**− 1**^	g kg^**− 1**^	% EI	g d^**− 1**^	g kg^**− 1**^	% EI	g d^**−1**^	g kg^**− 1**^	% EI
**HCLF**	2567 ± 463	34.1 ± 3.4	430 ± 82.6	5.7 ± 0.6	68.6 ± 1.4%	42.3 ± 8.5	0.6 ± 0.1	15.5 ± 1.1%	98.3 ± 14.3	1.3 ± 0.1	16.0 ± 1.2%
**LCHF**	2611 ± 436	34.7 ± 3.0	100 ± 23.8	1.3 ± 0.2	15.6 ± 1.8%	185 ± 30.1	2.5 ± 0.2	66.7 ± 2.3%	110 ± 19.1	1.5 ± 0.2	17.6 ± 1.2%
***p***	0.363		***< 0.001***			***< 0.001***			***0.013***		

**Fig. 2:**
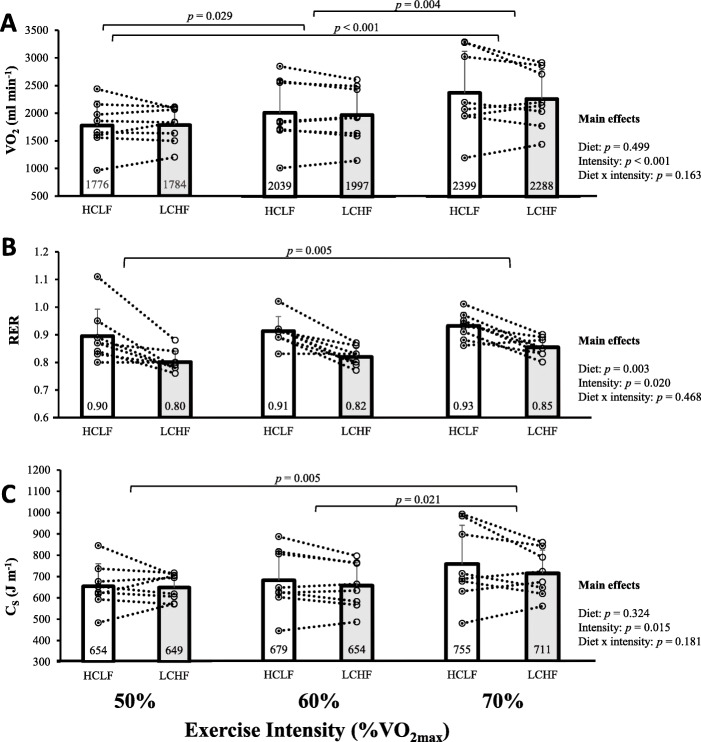
Rate of oxygen consumption (VO_2_, panel **a**), respiratory exchange ratio (RER, panel **b**), and energetic cost of swimming (C_S_, panel **c**) at swimming velocities corresponding to approximately 50, 60, and 70% of maximal aerobic capacity (VO_2max_) following 3 days of high-carbohydrate, low-fat (HCLF) and low-carbohydrate, high-fat (LCHF) diets. Bar graphs denote mean responses and error bars denote standard deviations for 8 subjects. Data points connected by dotted lines denote individual responses

Relative swimming intensity during each submaximal stage corresponded to 56.7 ± 1.1%, 63.1 ± 0.3%, and 73.6 ± 1.1% VO_2max_ for stages 1, 2 and 3, respectively, with no differences between diets (*p* = 0.970). Actual swimming speeds were 0.95 ± 0.10 m s^− 1^, 1.05 ± 0.11 m s^− 1^, and 1.12 ± 0.10 m s^− 1^. Rate of O_2_ consumption increased with exercise intensity (*F*(1.18,8.23) = 23.79, *p* <  0.001), but there was no difference between diets (Fig. 2a). Participants had a higher RER following the HCLF diet (*F* [1, 7] = 19.59, *p* = 0.003) and RER differed with exercise intensity (*F*(1.20,8.43) = 7.67, *p* = 0.020, Fig. 2b). Energetic cost of swimming ranged from 649 J m^− 1^ at 50% VO_2max_ on the LCHF diet to 755 J m^− 1^ at 70% VO_2max_ on the HCLF diet. There was a significant effect of exercise intensity (*F*(1.21,8.48) = 8.49, *p* = 0.015), but not diet (*F* [1, 7] = 1.12, *p* = 0.324), on C_s_ (Fig. 2c).

**Fig. 3:**
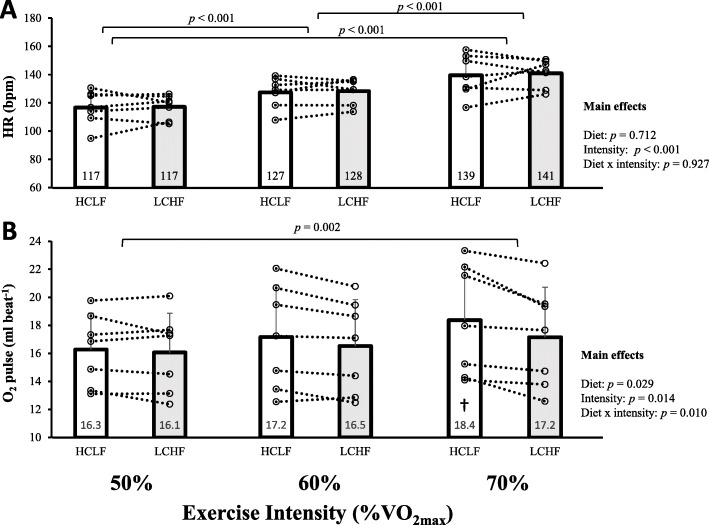
Heart rate (HR, panel **a**) and oxygen pulse (panel **b**) at swimming velocities corresponding to approximately 50, 60, and 70% of maximal aerobic capacity (VO_2max_) following 3 days of high-carbohydrate, low-fat (HCLF) and low-carbohydrate, high-fat (LCHF) diets. Bar graphs denote mean responses and error bars denote standard deviations for 7 subjects (one subject omitted due to technical difficulties with HR measurement). Data points connected by dotted lines denote individual responses. † denotes significantly different from HCLF 50% and different from LCHF at all intensities (*p* < 0.02)

One female participant’s HR monitor malfunctioned during the LCHF testing, so her data was excluded from all HR and O_2_ pulse aggregate data. Heart rate increased with exercise intensity (*F* [2, 12] = 59.7, *p* <  0.001), but there were no differences in HR between diets (*F* [1, 6] = 0.15, *p* = 0.712, Fig. 3a). Oxygen pulse was greater in the HCLF diet (*F* [1, 6] = 8.17, *p* = 0.029), with significant main effects of exercise intensity (*F*(1.16, 6.99) = 10.02, *p* = 0.014), and a significant interaction between diet and intensity (*F* [2, 12] = 7.03, *p* = 0.010, Fig. 3b).

## Discussion

The purpose of this study was to determine the influence of a 3-day, high- or low-carbohydrate diet on swimming economy in recreationally-trained swimmers. It was hypothesized that the HCLF diet would increase carbohydrate utilization relative to a LCHF diet, resulting in an increase energy conversion per volume of O_2_ consumed. Although RER was greater during exercise following 3 days of a HCLF diet, no differences in VO_2_ or C_s_ were detected between diets. Therefore, these data do not support our original hypothesis because there was no improvement in swimming economy (i.e., a reduction in C_s_) with the HCLF diet. To our knowledge, this is the first study investigating the effects of diet on swimming economy.

Results from the present study are in contrast with previous research reporting an effect of diet on movement economy in cyclists, runners, and race walkers [7, 8, 12–15, 24], though comparisons across studies are challenged by differences in study design. Using a dietary protocol similar to our study, Cole et al. [12] reported increased cycling efficiency following a 3-day high-carbohydrate diet (70% energy intake), compared with consuming 20% or 45% carbohydrate. Additionally, a 5-day ketogenic LCHF diet decreased economy in race walkers [7, 8]. Studies using longer dietary interventions (e.g. 3–4 weeks) have also reported decreases in movement economy following ketogenic LCHF diets [13–15]. Due to the confounding effects of ketone oxidation on RER [19], it is unclear how ketogenic vs. non-ketogenic LCHF diets influence fat oxidation and exercise economy. However, the contribution of ketone oxidation to energy production in skeletal muscle appears small when other substrates are available [25]. Taken together, the duration and type of LCHF diet used in our study are unlikely to be the reason for the lack of detectable changes in swimming economy.

Differences in mode of exercise may explain the lack of effect of diet in the present study. Aggregate C_S_ in our study ranged from 649 to 755 J m^− 1^, which is similar to values reported in other swimming studies [26, 27]. In comparison, the energetic cost of running and race walking are approximately half of C_S_ values reported in the present study [13, 14]. For swimming, minimizing drag force and maximizing propelling efficiency (i.e.*,* maximizing useful power and minimizing wasted power) are adaptations of higher-caliber swimmers that improves swimming economy [28]. In addition, swimming technique likely plays a greater role in swimming economy than cycling or running technique do for economy in those sports because the swimmer moves through water. Therefore, any diet effects on swimming economy may have been masked by slight changes in stroke mechanics between trials, though mechanics were neither measured nor controlled.

Our contrasting findings could also be related to the exercise intensities used in our study, which were lower than what has been used in previous studies [7, 8, 14, 15]. Therefore, subjects in the present study were more reliant on fat oxidation. In support of this hypothesis, Shaw et al. [13] reported that running economy was impaired at intensities over 70% VO_2max_, but preserved at intensities lower than 60% VO_2max_, following 31 days of a ketogenic LCHF diet in trained runners. Additionally, 4 weeks of a LCHF diet had no impact on cycling economy in endurance-trained athletes cycling at ~ 63% VO_2max_ [29]. Movement economy was impaired by LCHF diets between 64 and 90% VO_2max_ in elite male race walkers [7, 8, 14, 15] and recreationally-trained male runners [30], and improved by a HCLF diet at 70–75% VO_2max_ in trained male cyclists [12]. However, decreased cycling efficiency following LCHF has also been reported at ~ 40–45% VO_2max_ in untrained males [24].

Oxygen pulse was higher following the HCLF diet. Although body weight and hydration status were not measured in this study, it is likely that the HCLF diet caused an increase in total body water due to increases in muscle glycogen [31]. Water stored with muscle glycogen would increase intracellular but not extracellular water content [32], though it is possible that this extra water may have played a role in the increased oxygen pulse observed during submaximal exercise in following the HCLF diet. A short-term (5–6 d) LCHF diet can lead to slower VO_2_ kinetics, concomitant with slower and reduced activation of mitochondrial pyruvate dehydrogenase [24, 33], but in the present study we found VO_2_ stabilized between 2 and 3 min into each 5-min stage (*data not shown*). This indicates subjects were below their critical swimming velocity, above which VO_2_ and lactate will not stabilize [34].

For many of the participants, this study was their first experience swimming in a flume. Subjects received a familiarization swim prior to each trial, gained experience in the flume from their initial VO_2max_ test, and the order of the diets was randomized so any learning effects of swimming in the flume should not affect conclusions from this study. A strength of this study is that the diets were individually tailored for each participant, based on their habitual food choices. Participants were required to shop for and prepare all of their own food in the quantities specified, and could communicate with the researchers about food choices and potential substitutions if needed. This design increases the ecological validity of this study as most recreational swimmers do not have access to the same resources as collegiate and professional swimmers whose diet and exercise volume can be more closely monitored. It is possible that participants could have been untruthful in recording their diets, although the difference in RER between diets suggests acceptable adherence. Protein intake was significantly different between groups (16.0 for HCLF vs. 17.6% for LCHF), but this difference is likely too small to impact the results of this study.

## Conclusions

To our knowledge, this study was the first to examine the effects of macronutrient intake on swimming economy. The HCLF diet increased carbohydrate utilization but did not improve swimming economy compared to the LCHF diet. Therefore, a LCHF diet may offer potential benefit for ultra-distance swimmers, who compete in long-duration, low-intensity events during which consuming large quantities of carbohydrate is challenging. Future research should focus on how diet affects swimming economy during long-duration swims where combating fatigue and maintaining swimming economy are important for success in marathon and ultramarathon swimming events.

## Data Availability

The datasets used and/or analyzed during the current study are available from the corresponding author on reasonable request.

## Abbreviations

HCLF: High-carbohydrate, low-fat
LCHF: Low-carbohydrate, high-fat
VO_2max_: Maximal aerobic capacity
VCO_2_: Rate of CO_2_ production
VO_2_: Rate of O_2_ consumption
RER: Respiratory exchange ratio
EE: Rate of energy expenditure
C_S_: Energetic cost of swimming
